# The complete mitogenome of *Fusarium equiseti*

**DOI:** 10.1080/23802359.2019.1661298

**Published:** 2019-09-05

**Authors:** Xiaotang Sun, Mengshuang Shu, Pengmei Shang, Ruqiang Cui

**Affiliations:** College of Agronomy/Key Laboratory of Crop Physiology, Ecology and Genetic Breeding, Ministry of Education, Jiangxi Agricultural University, Nanchang, Jiangxi, China

**Keywords:** *Fusarium equiseti*, mitogenome, phylogenetic analysis

## Abstract

The complete mitochondrial genome of plant pathogenic fungus, *Fusarium equiseti*, was sequenced. The circular molecule is 53,411 bp long with a GC content of 32.81%. It contains 22 protein-coding genes, 4 ribosomal RNA (rRNA), and 24 transfer RNA (tRNA) genes. Phylogenetic reconstructions confirmed that it has the closest relationship with *Fusarium equiseti*. The mitogenome analysis of *Fusarium equiseti* provides a molecular basis for further studies on molecular systematics and evolutionary dynamics.

The significantly important plant pathogens of species belonging to the genus *Fusarium* cause diseases in many crops and wild plants. The isolate of *Fusarium equiseti* is a strongly virulent strain, which can cause leaves wilting. We sequenced the complete mitogenome (mitochondrial genome) of the *Fusarium equiseti* strain 2018BL08 (GenBank accession number MN199625) isolated from *Nelumbo nucifera* in Guangchang City of Jiangxi Province (26 47′49ʺ N, 116_18′11ʺ E) and the specimens were stored at the Plant Pathology Lab in Jiangxi Agricultural University. DNA isolation using an improved extraction method (Chen et al. 2011) and libraries were sequenced on the Illumina Hiseq 4000 (Borgström et al. 2011) (Shanghai BIOZERON Co., Ltd, Shanghai, China) with a 150 bp paired-end read.

The filtered reads were assembled using ABySS (Simpson et al. 2009) and verifying the assembly and completing the circle. ORF Finder (http://www.ncbi.nlm.nih.gov/gorf/gorf.html) was used to identify any potential genes in the uncoding regions. The mitochondrial genes were annotated using homology alignments and de novo prediction. Transfer RNA (tRNA) genes and ribosome RNA (rRNA) genes were predicted by tRNAscan-SE (Lowe and Eddy 1997) and rRNAmmer 1.2 (Lagesen et al. 2007).

The genome has a total length of 53,411 bp and the nucleotide composition of the mitogenome is: 34.05% of A, 33.13% of T, 17.81% of G, and 15.01% of C. It contains 22 protein-coding genes, 4 ribosomal RNA (rRNA), and 24 transfer RNA (tRNA) genes. The tRNA genes contain codons for all 20 standard amino acids. Most amino acids are represented by only one tRNA gene, however, two trnL (trnL-UAA and trnL-UAG), two trnG (trnG-ACC and trnG-UCC), two trnR (trnR-ACG and trnR-UCU), and two trnS (trnS-GCU and trnS-UGA) genes are found in this mitochondrial genome.

Nine members of Nectriaceae are included in the phylogenetic analysis, including eight taxa of *Fusarium*. Maximum likelihood (ML) were used to construct the phylogenetic trees with all protein-coding genes and rRNA by PhyML v3.0 (http://www.atgc-montpellier.fr/phyml/) (Liu et al. 2019).

As shown in Figure 1, *Fusarium graminearum* (HG970331), *Fusarium gerlachii* (KM486533), and *Fusarium culmorum* (KP827647) are determined as sisters of *Fusarium equiseti* with strong support. High bootstrap and posterior probability values show that presented relations are stable. The mitochondrial genome of *Fusarium equiseti* will contribute to the understanding of phylogeny.

**Figure 1.**
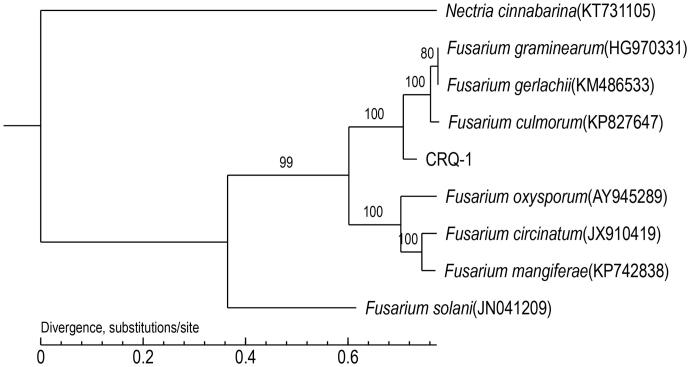
Phylogenetic analysis of 9 members of Nectriaceae based on core protein-coding genes.
